# Echocardiographic Updates in the Assessment of Cardiomyopathy

**DOI:** 10.1007/s11886-024-02159-7

**Published:** 2025-01-22

**Authors:** Baoqiong Liu, Kandarp Suthar, Christine M. Gerula

**Affiliations:** 1 Division of Cardiology, Department of Medicine, Rutgers New Jersey Medical School, Newark, NJ USA; 2 Rutgers - New Jersey Medical School, 185 S Orange Ave, Newark, NJ 07103 USA

**Keywords:** Echocardiography, Cardiomyopathy, Strain imaging, 3D echocardiography, Artificial intelligence, Diagnostic advancements

## Abstract

**Purpose of Review:**

This review aims to provide an updated overview of the role of echocardiography in the assessment of cardiomyopathies, highlighting recent findings and technological advancements.

**Recent Findings:**

Over the past few years, significant advancements in echocardiographic techniques have improved diagnostic accuracy and provided important prognostic value in the assessment of cardiomyopathies.

**Summary:**

Cardiomyopathy is a group of diseases affecting the heart muscle. Echocardiography, a non-invasive imaging modality provides crucial information on cardiac structure, function, and hemodynamics. Recent advancements, including strain imaging, speckle-tracking, and 3D echocardiography enhance the precision of structural and functional assessments, while artificial intelligence integration improves diagnostic accuracy and workflow efficiency. These advancements not only refine diagnostic capabilities but also provide prognostic insights and facilitate better patient outcomes.

## Introduction

Cardiomyopathy is a group of diseases affecting the myocardium. It is defined as myocardial disorder in which the heart muscle is structurally and functionally abnormal, in the absence of coronary artery disease, hypertension, valvular disease, and congenital heart disease sufficient to cause the observed myocardial abnormality. Cardiomyopathies are morphologically categorized into dilated, hypertrophic, restrictive, and arrhythmogenic cardiomyopathies (ACM), and unclassified cardiomyopathy [1]. Echocardiography is a non-invasive, low-cost, and widely available imaging modality. It provides relevant information on global and regional right ventricle (RV) and left ventricle (LV) anatomy and function, valve function, and the presence of dynamic obstruction, pulmonary hypertension, or pericardial effusions. Echocardiography is routinely used in the diagnosis, risk stratification, and follow-up of patients with cardiomyopathy. This update explores the latest advancements for the assessment of cardiomyopathies.

## Evaluation of Specific Cardiomyopathies by Conventional Echocardiography

### Hypertrophic Cardiomyopathy (HCM)

**Fig. 1:**
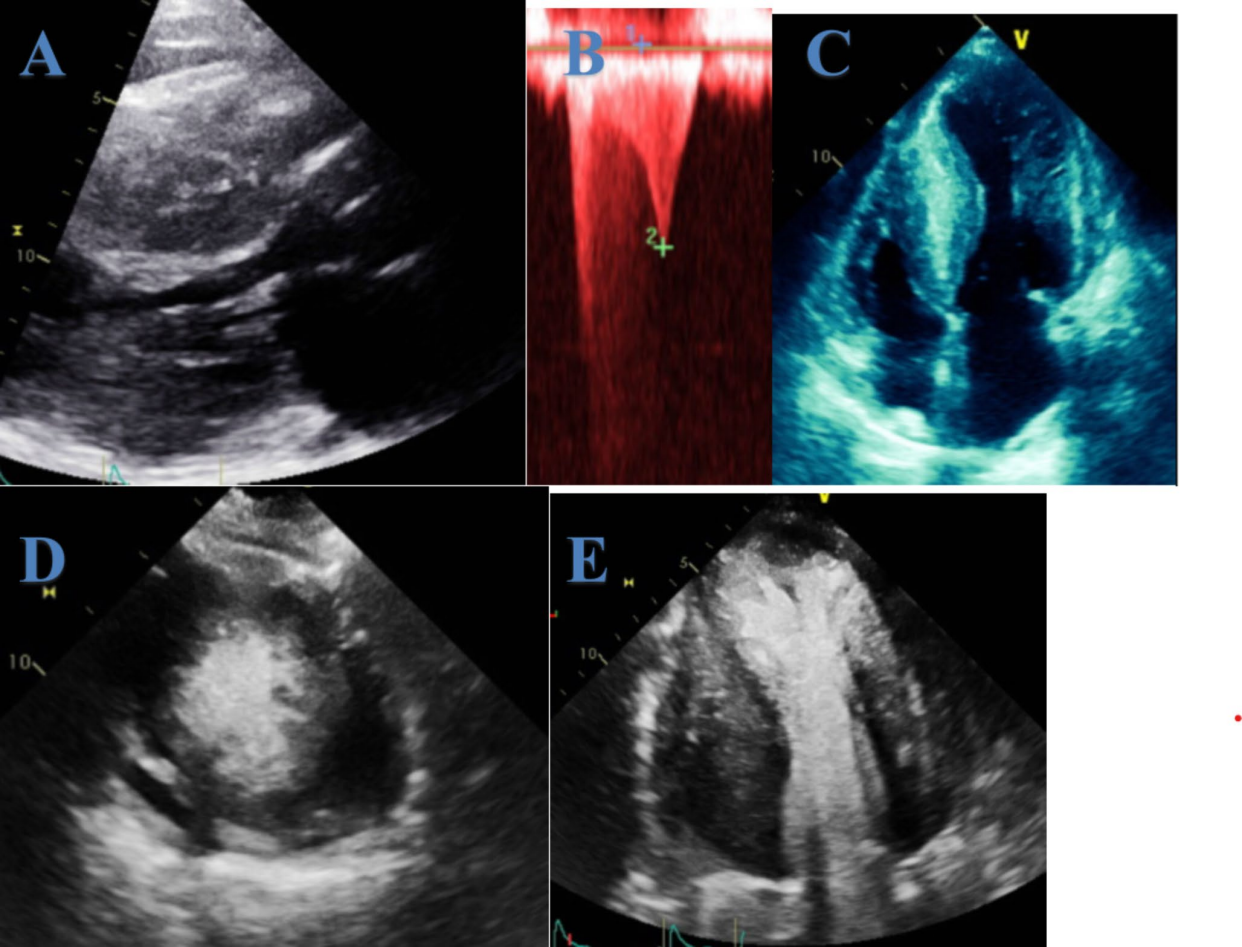
Echocardiography of a patient with hypertrophic obstructive cardiomyopathy. **A**: Parasternal long-axis view showing a thickened left ventricular wall. **B**: Doppler of the left ventricular outflow tract (LVOT) reveals a characteristic dagger-shaped signal with a LVOT gradient of 64 mmHg. **C**, **D**, and E: Thickened ventricular walls are shown, with and without contrast enhancement

HCM is defined by the presence of left ventricular hypertrophy, with or without LV outflow tract (LVOT) obstruction, in the absence of other potentially causative cardiac, systemic, syndromic, or metabolic diseases [2]. Transthoracic echocardiography (TTE) is the initial imaging modality of choice in diagnosing HCM. A wall thickness ≥ 15 mm in the absence of other causes of hypertrophy in a non-dilated left ventricle (LV) defines HCM (Fig. 1). In cases with a family history of HCM, a wall thickness ≥ 13 mm can be considered diagnostic [2]. Studies have shown that the median maximal wall thickness measured by echocardiography is lower than that measured by cardiac magnetic resonance imaging (cMRI) in HCM patients, with echocardiography-based estimates of sudden cardiac death risk being 8.33% lower than MRI-based estimates [3]. While most patients with HCM have normal to hyperdynamic left ventricular ejection fraction (LVEF), LV dysfunction (defined as LVEF < 50%) occurs in about 8% of cases in a large international cohort, and is associated with higher rates of all-cause mortality, cardiac transplantation, and the need for left ventricular assist devices [4, 5].

Diastolic dysfunction occurs in HCM patients due to impaired LV relaxation and increased myocyte and chamber stiffness [6]. Impaired relaxation can be detected in young carriers of sarcomere gene variants who have normal LV wall thickness, indicating that diastolic abnormalities may be an early sign of pre-clinical HCM [7]. While diastolic dysfunction is rarely observed in young elite athletes (≤ 0.5%), septal early diastolic myocardial velocity (E′) < 10 cm/sec and lateral E’ < 12 cm/sec best differentiate HCM from athlete’s heart [8]. Restrictive LV filling pattern and increased early diastolic filling velocity (E) to E’ (E/E’) ratio have been associated with heart failure hospitalizations, reduced exercise tolerance, myectomy, and cardiac death in patients with HCM [9, 10]. Additionally, systolic-diastolic coupling is impaired in HCM patients and is associated with fitness and the cardiac response to exercise [11]. Apical HCM should be suspected when there is deep T-wave inversion on an electrocardiogram, along with a thickened LV apex that displays an ace-of-spades appearance during diastole [12].

LVOT obstruction is considered present if the peak LVOT gradient is ≥ 30 mm Hg. Resting or provoked gradients ≥ 50 mm Hg are regarded as sufficient to cause symptoms [6]. Because LVOT gradients vary with heart rate, blood pressure, volume status, activity, and medications, provocative maneuvers such as standing, Valsalva strain, or exercise are sometimes needed to make the diagnosis of LVOT obstruction by echocardiography [13, 14]. Eating before echocardiography has also been found to be a powerful provocative tool, as postprandial echocardiography unmasks LVOT obstruction in more than one-third of patients who do not have high gradients otherwise [15]. However, using Dobutamine to identify latent LVOT obstruction and eligibility for advanced therapies is not recommended due to a lack of specificity [6]. Echocardiographic criteria to determine eligibility for implantable cardioverter-defibrillator (ICD) include massive LV hypertrophy ≥ 30 mm in any LV segment and LV systolic dysfunction (EF < 50%) [16]. For patients suspected of having HCM in whom TTE is inconclusive or not diagnostic, cMRI imaging can aid with diagnosing HCM [17].

### Dilated Cardiomyopathy (DCM)

Dilated cardiomyopathy (DCM) is characterized by LV dilatation and global or regional systolic dysfunction, not solely explained by abnormal loading conditions such as hypertension or valve disease, and it can be a rare result of cardiac sarcoidosis. In DCM, echocardiography is essential for assessing ventricular size, function, and morphology. LV dilatation is defined as LV end-diastolic volume index > 96 mL/m^2^ for females and > 105 mL/m^2^ for male patients [18]. Isolated LV dilatation with normal ejection fraction may represent an early manifestation of DCM. Right ventricular dilatation and dysfunction may also be present in patients with DCM and are an adverse prognostic marker. Left atrial volume index (LAVi) above 50 mL/m2 is a characteristic echocardiographic feature of DCM. In a recent study, LV diastolic function was evaluated according to the 2016 American Society of Echocardiography in patients with DCM, and all patients had LV diastolic dysfunction, with grade II diastolic dysfunction (44.6%) and grade III (35.8%) accounting for the majority [19].

It is widely acknowledged that LV end-systolic volume and LVEF predict worse long-term survival in DCM patients. The myocardial performance index (MPI/Tei Index), which measures the combined systolic and diastolic function, is calculated by adding the isovolumic contraction time and isovolumic relaxation time and then divided by the ejection time. The Tei index is associated with mortality in patients with DCM, with those having a high Tei index (>/=0.76) experiencing shorter life expectancy [20]. Additionally, a high echocardiography-derived hemodynamic forces angle is associated with major adverse cardiovascular events (MACE) in DCM patients and may serve as a marker of impaired myocardial performance in patients with reduced LVEF [21]. In a recent study, RV function, assessed by fractional area change (FAC), tricuspid annular plane systolic excursion (TAPSE), RV longitudinal strain, and RV size were found to be independent predictors of LV assist device implantation and all-cause death within one year in patients with DCM [22]. Repeat echocardiography, 40 days after myocardial infarction 90 days after revascularization, and 90 days after guideline-directed medical therapy (GDMT), plays a pivotal role in evaluating candidacy for implantable cardioverter defibrillator (ICD) or cardiac resynchronization therapy (CRT) [23]. 2022 AHA/ACC/HFSA guidelines for heart failure management recommend ICD for patients with LVEF ≤ 35% and New York Heart Association (NYHA) class II or III symptoms on chronic GDMT; or with LVEF ≤ 30% and NYHA class I symptoms while receiving GDMT [23].

### Restrictive Cardiomyopathy (RCM)

**Fig. 2:**
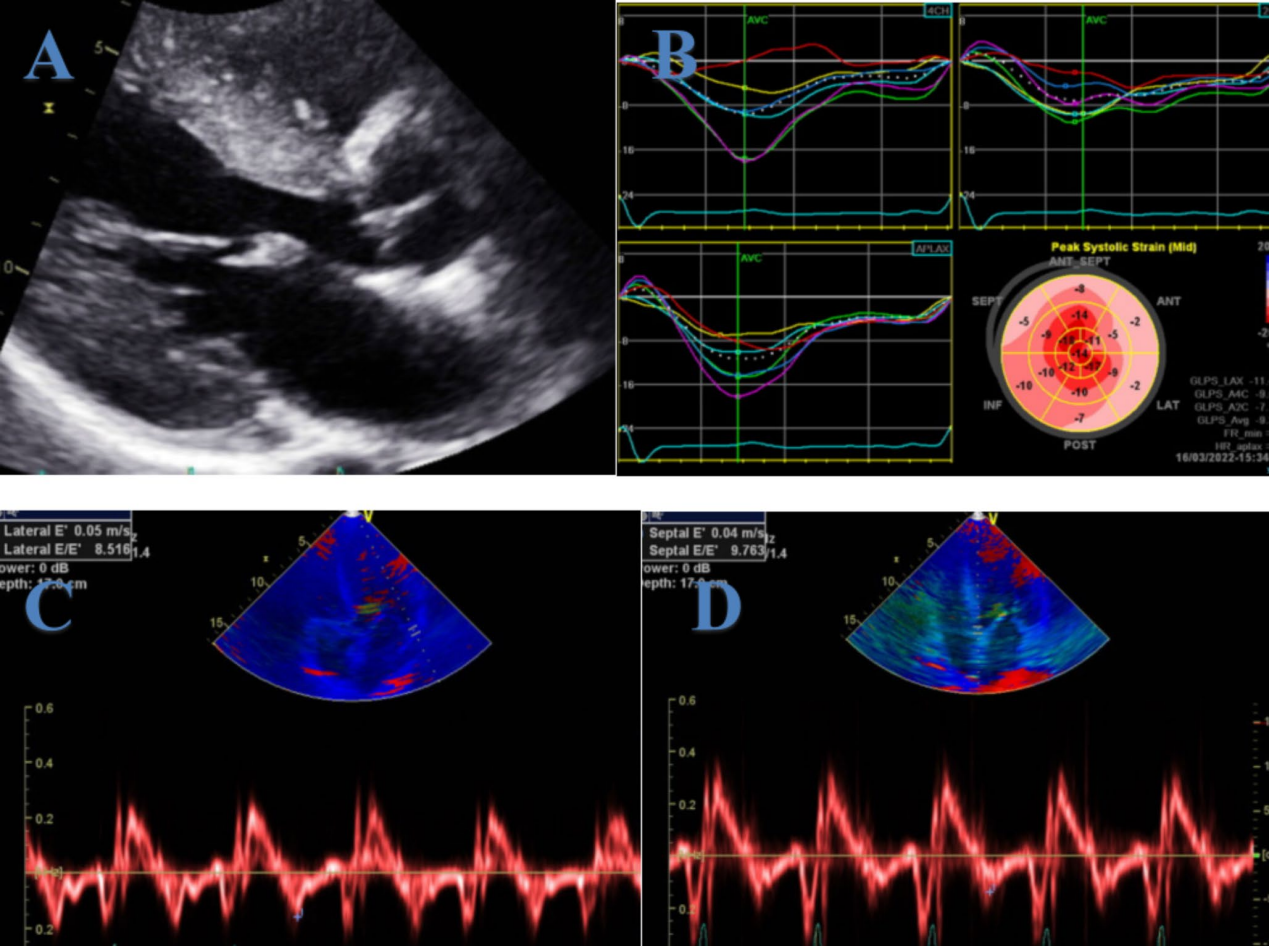
Echocardiography of a patient with cardiac amyloidosis. **A**: Parasternal long-axis view showing a thickened left ventricular wall. **B**: Global longitudinal strain of apical sparing pattern. **C** and **D**: Tissue doppler consistent with decreased diastolic function

Restrictive cardiomyopathy (RCM) comprises a heterogeneous group of diseases characterized by restrictive ventricular pathophysiology [24]. Patients with RCM exhibit a rigid, noncompliant left ventricle (LV) with impaired diastolic filling and elevated filling pressures. The initial clue of RCM in echocardiography is the presence of biatrial enlargement, which cannot be attributed to specific causes such as valve disease or atrial fibrillation, combined with non-dilated ventricles [24]. Chronically elevated LV diastolic pressures often lead to pulmonary hypertension, which can cause or exacerbate right heart failure. While LV systolic function is typically preserved in the early stages of RCM, it tends to deteriorate over time. Ventricular wall thickness varies with the etiology of RCM. Wall thickness is often normal in hemochromatosis, increased in cardiac amyloidosis (Fig. 2), Fabry’s disease, and glycogen storage disorders, and can be decreased with thinning of the ventricular septum in patients with sarcoidosis [25].

RCM Doppler imaging can show a restrictive filling pattern of transmitral flow with an increased E to atrial filling velocity ratio. The ratio between systolic and diastolic pulmonary venous flow ratios can be markedly reduced due to high LA pressures [26]. Tissue Doppler typically shows reduced E′ [26], which differentiates RCM from constrictive pericarditis [27, 28]. Hepatic veins diastolic flow reversal during inspiration is common due to the inability of a non-compliant RV to accommodate the increased venous return, as compared to expiatory reversal in patients with constrictive pericarditis with a hepatic vein ratio in expiration ≥ 0.79 [29].

### Arrhythmogenic Cardiomyopathy

Arrhythmogenic cardiomyopathy (ACM) is an inherited heart muscle disease characterized by progressive fibro-fatty replacement of the myocardium [30]. The main clinical manifestation of ACM is the occurrence of malignant ventricular arrhythmias. The echocardiographic characteristics of ACM have been reported in previous studies, including increased right ventricular dimensions, decreased RV systolic function, and right ventricular morphologic abnormalities. However, LV can be involved in other clinical variants. The 2020 International Expert Consensus document Padua Criteria for diagnosis of ACM requires detecting at least one major or minor criterion in either morpho-functional ventricular abnormalities or structural myocardial abnormalities. Key indicators are segmental akinesia, dyskinesia, and aneurysms, which help in differentiating it from other cardiomyopathies [31].

Echocardiographic parameters have been shown to predict adverse cardiac outcomes in ACM. Tricuspid and septal S’ by tissue Doppler imaging has been associated with ventricular arrhythmia. Right atrial diameter on echocardiography, along with RVEF and LVEF by cardiac MRI, are predictors of cardiac death and the need for heart transplantation [32]. Patients with structural progression detected by echocardiography are at higher risk of MACE; 64% of patients with MACE had structural progression [33], highlighting the importance of regular imaging follow-up. Both RV and LV function characterized by echocardiogram serve as important criteria for primary ICD implantation in the ACM population. According to 2015 International Task Force Consensus on the treatment of ACM, there is a Class I indication for ICD in patients with severe RV dysfunction (RV fractional area change ≤ 17% or RV EF ≤ 35%) or LV dysfunction (LV EF ≤ 35%) and Class IIa indication for patients with moderate RV dysfunction (RV fractional area change between 24 and 17% or RV EF between 40 and 36%), or LV dysfunction (LV EF between 45 and 36%) [34].

## Strain Imaging and Speckle-Tracking Echocardiography

Deformation imaging (strain) measures the fractional change in length of a segment of the myocardium in longitudinal, circumferential, and radial directions during the cardiac cycle. The technique involves generating speckles that represent distinct myocardial segments, which are tracked frame by frame throughout the cardiac cycle. Speckle tracking with global longitudinal strain (GLS) is a more sensitive marker for detecting left ventricular dysfunction [35, 36]. It can demonstrate a range of regional and global abnormalities even in patients with normal wall motion and LVEF and has superior interobserver and intraobserver reproducibility compared to ejection fraction [37].

Strain imaging can help discriminate between different etiologies of hypertrophy [38]. Left ventricular GLS was higher in athletes than in patients with HCM and hypertensive heart disease, with the lowest LV-GLS value in the HCM group; and severely reduced LV-GLS below − 12.5% was associated with all-cause mortality [39]. Apical Sparing and apical-to-basal segmental longitudinal strain of > 2.1 differentiate cardiac amyloidosis from other causes of concentric LV hypertrophy. In the bull’s eye map, a strain pattern similar to stripes was identified in 60% of patients, which might be a differentiating marker of Glycogen storage cardiomyopathy [40].

Recent studies have highlighted the prognostic value of strain imaging. The worsening of RV GLS is associated with an increased risk of adverse cardiac events and poor treatment response in patients with dilated cardiomyopathy [41]. LV GLS has been found to predict composite cardiac outcomes and ventricular arrhythmias in patients with hypertrophic cardiomyopathy [42, 43]. Global work index (GWI), calculated as the area of the LV pressure-strain loop, and global constructive work - a measure of the positive work performed by the LV segments during systolic shortening, and the negative work performed during lengthening in the isovolumic diastole, have an additional value beyond LV-EF and GLS for predicting adverse outcomes in DCM [44]. Reduced RV global longitudinal strain and RV free wall strain, which are not part of the 2020 Padua criteria, can be used as additional parameters in diagnosing ACM [45]. Mechanical dispersion, defined as the standard deviation of the time-to-peak longitudinal strain of all segments, is a marker of contraction inhomogeneity and highlights fine structural changes that may be missed by other modalities. Mechanical dispersion (MD) has been found to independently predict ventricular arrhythmia. Patients with nonischemic cardiomyopathy and MD > 70 ms had high arrhythmic event rate, comparable to the general population [46].

Strain imaging enables early detection of disease expression in relatives of patients with a genetic cardiomyopathy [47], possibly reflecting early cardiomyocyte loss in DCM, myocardial disarray and interstitial fibrosis in HCM, and desmosomal dysfunction in ACM [36]. Multiple deformation parameters including peak left atrial longitudinal strain [48] have been studied in predicting the development of genetic cardiomyopathies in relatives. The key findings reproduced by different studies show reduced LV GLS in relatives of DCM patients are at risk for developing DCM despite standard echocardiographic parameters being within the normal range [47, 48], Additionally, reduced basal anteroseptal strain in relatives of HCM patients predicts the development of HCM. Relatives of ACM patients with reduced RV-free wall strain are also at risk for developing ACM [49, 50].

Echocardiographic strain measurements can have significant intervendor and interobserver variability. A vendor-independent, open-source, deep learning-based strain algorithm has been studied for measuring LV GLS, showing lower variation compared to human measurements while yielding similar quantitative results [51].

## 3D Echocardiography

One major advantage of three-dimensional (3D) echocardiography is that it allows for more accurate and reproducible measurements of cardiac chamber volumes and LV ejection fraction compared with 2D echocardiography, by eliminating the need for geometric modeling and the errors caused by foreshortened apical views [52]. 3D echocardiography has been identified as the best echocardiographic method for sequential quantification of LV volumes and ejection fraction in patients undergoing chemotherapy [53]. Optimized 3D automated techniques, designed to resemble the magnetic resonance reference and can further improve accuracy and have been found to measure LV volumes with less than 10% inter-measurement variability [54].

In patients with ACM, 3D echocardiography is more sensitive in identifying RV wall motion abnormalities than two-dimensional echocardiography, with a detection rate comparable to cMRI, although it underestimates RV volumes in comparison with cMRI [55]. A 3D RVEF lower than 43.4% has been identified as an independent predictor of major adverse cardiovascular events in patients with DCM [56]. Using 3D echocardiography, it has been observed that atrial fibrillation induces significant remodeling in all heart chambers in patients with DCM [57]. Additionally, 3D echocardiography is highly effective in anatomical analysis of the interventricular septum and abnormal muscle bundles for septal myectomy planning of HCM patients [58].

3D global area strain (GAS) is the percentage decrease in the size of the LV’s endocardial surface area from end-diastole to end-systole. 3D GAS and 3D LV GLS are advantageous over 2D LV GLS for evaluating myocardial function, because speckles can be tracked in 3D space irrespective of the motion direction, however, it is not feasible in one-third of patients [59]. 3D GLS may serve as an optimal surrogate marker for reflecting myocardial fibrosis in patients with dilated cardiomyopathy with advanced heart failure [60]. Impaired 3D GLS has been associated with worse functional capacity, as assessed by peak oxygen consumption and higher NYHA classes in patients with HCM [61]. There is a possible linear correlation between conventional risk markers of sudden cardiac death and 3D deformation parameters in HCM [62]. Regional shape indices by 3D can provide additional prognostic value [63].

### Artificial Intelligence and Machine Learning

The application of artificial intelligence (AI) in echocardiography is rapidly expanding. AI has been reported to enhance workflow efficiency, improve accuracy, inter-reader agreement, and increase reader confidence [64]. AI-based algorithms facilitate view recognition, segmentation, and analysis of conventional measurements, thus reducing data acquisition time [65]. AI-assisted assessments of LVEF [66] and global longitudinal strain (GLS) [67] are highly reproducible. In a large, blinded, randomized clinical trial, AI-based LVEF assessments outperformed those by sonographers, were less likely to be changed by cardiologists, took less time to overread, and were more consistent with previous clinical reports [68]. A recently developed deep learning algorithm enables fully automated interpretation of echocardiographic data, cutting the analysis time to under five minutes [69].

AI models based on echocardiography videos have been trained to detect cardiomyopathies, with diagnostic accuracy being comparable to the interpretation by human experts in differentiating cardiac sarcoidosis from healthy subjects [70]. Machine-learning algorithms developed from speckle-tracking echocardiographic datasets can assist in discriminating between physiological and pathological patterns of hypertrophic remodeling [71]. An apical four-chamber view-based AI model can effectively distinguish between constrictive pericarditis and restrictive cardiomyopathy, with a diagnostic area under the curve of 0.97 [72]. A 3D convolutional neural network trained on apical 4-chamber video demonstrates excellent discrimination of patients with versus without heart failure with preserved EF [73]. An automated AI strategy developed using electrocardiograms and echocardiograms can augment cardiac amyloidosis detection [74]. Another AI model can differentiate atrial septal defect, dilated cardiomyopathy, hypertrophic cardiomyopathy, and prior myocardial infarction from normal subjects [75]. This AI framework classifies echocardiographic videos into five diagnostic categories by first extracting features from frames using a retrained Inception-V3 network and then analyzing these features with a diagnostic neural network. It identifies anatomic regions of interest relevant to each diagnosis, and its performance was comparable to that of the consensus of three senior cardiologists [75].

## Conclusions

Echocardiography remains an essential tool in the diagnosis, risk stratification, and follow-up of cardiomyopathies. Recent advancements in echocardiographic techniques, including strain imaging, 3D echocardiography, and artificial intelligence, have significantly enhanced our ability to assess cardiac structure and function in these patients. Strain imaging provides valuable insights into myocardial deformation and has been shown to predict adverse cardiac events in various cardiomyopathies. 3D echocardiography offers improved accuracy in measuring cardiac chamber volumes and assessing myocardial morphology. Artificial intelligence has the potential to streamline the echocardiographic workflow, improve diagnostic accuracy, and enable automated analysis of echocardiographic data. By incorporating these advanced tools into clinical practice, we can potentially improve the diagnosis and management of cardiomyopathies, leading to better patient outcomes.

## Key References

- Marstrand P, Han L, Day SM, Olivotto I, Ashley EA, Michels M, et al. Hypertrophic Cardiomyopathy With Left Ventricular Systolic Dysfunction: Insights From the SHaRe Registry. Circulation. 2020;141 [17]:1371-83.
-
  - This study showed LV dysfunction with EF < 50% happens in 8% of HCM patients and is associated with worse outcomes.
- Abou Alaiwi S, Roston TM, Marstrand P, Claggett BL, Parikh VN, Helms AS, et al. Left Ventricular Systolic Dysfunction in Patients Diagnosed With Hypertrophic Cardiomyopathy During Childhood: Insights From the SHaRe Registry. Circulation. 2023;148 [5]:394–404.
-
  - This study demonstrated patients with childhood-diagnosed HCM have a significantly higher lifetime risk of developing LV systolic dysfunction than for patients with adult-diagnosed HCM.
- MacNamara JP, Turlington WM, Dias KA, Hearon CM, Jr., Ivey E, Delgado VA, et al. Impaired longitudinal systolic-diastolic coupling and cardiac response to exercise in patients with hypertrophic cardiomyopathy. Echocardiography. 2024;41 [6]:e15857.
-
  - This study showed systolic-diastolic coupling is impaired in HCM patients and is associated with cardiac response to exercise.
- Mutluer FO, Bowen DJ, van Grootel RWJ, Roos-Hesselink JW, Van den Bosch AE. Left ventricular strain values using 3D speckle-tracking echocardiography in healthy adults aged 20 to 72 years. Int J Cardiovasc Imaging. 2021;37 [4]:1189 − 201.
-
  - This study showed 3D strain is more accurate than 2D strain however is not feasible in one third of patients.
- Cardoso I, Viegas JM, Rosa SA, Bras PG, Grazina A, Cruz I, et al. Three-dimensional echocardiography for the evaluation of hypertrophic cardiomyopathy patients: relation to symptoms and exercise capacity. Int J Cardiovasc Imaging. 2023;39 [12]:2475-81.
-
  - This study showed 3D GLS has been associated with worse functional capacity in patients with HCM.
  - He B, Kwan AC, Cho JH, Yuan N, Pollick C, Shiota T, et al. Blinded, randomized trial of sonographer versus AI cardiac function assessment. Nature. 2023;616(7957):520-4.
-
  - This trial demonstrated AI-based LVEF assessments outperformed those by sonographers.
- Chao CJ, Jeong J, Arsanjani R, Kim K, Tsai YL, Yu WC, et al. Echocardiography-Based Deep Learning Model to Differentiate Constrictive Pericarditis and Restrictive Cardiomyopathy. JACC Cardiovasc Imaging. 2024;17 [4]:349 − 60.
-
  - This study developed an apical four chamber view based AI model, which can effectively distinguish between constrictive pericarditis and restrictive cardiomyopathy.

## Data Availability

No datasets were generated or analysed during the current study.
